# Gender transition during a clinical trial

**DOI:** 10.1016/j.jdcr.2024.03.010

**Published:** 2024-03-26

**Authors:** Joel L. Cohen, Sami I. El-Qadi

**Affiliations:** a Galderma, Lausanne, Switzerland; b AboutSkin Dermatology and DermSurgery, Greenwood Village and Lone Tree, Colorado; c Department of Dermatology, University of California Irvine, Irvine, California; d Galderma Laboratories, L.P., Fort Worth, Texas

**Keywords:** botox, botulinum toxin, clinical trials, controlled trials, cosmetic/aesthetic, gender identity, general dermatology, sexuality

In a recent multicenter, open-label, interventional study evaluating subject satisfaction with abobotulinumtoxinA for moderate to severe glabellar lines, a male subject transitioned to female during the course of the study.^1^ We believe this may be the first gender, or chosen sexual identitiy, transition in an aesthetic clinical trial in which the subject entered the study not receiving any hormonal therapy or indicating plans for gender-affirming care. The subject, a 28-year-old White man at enrollment, had previously received toxin treatments but expressed neither interest in nor plans for gender transition.

Baseline evaluation by the investigator (using a 4-point scale) revealed moderate glabellar lines at animation (grade 2) (Fig 1). At this baseline visit, the subject expressed dissatisfaction with their appearance—believing they looked older than their actual age. Furthermore, the lines between their eyebrows caused them moderate to extreme distress when contracting their facial muscles. The subject received a 50 U injection of abobotulinumtoxinA. The injection followed a 5-point pattern, with each injection site receiving 10 U of the neuromodulator agent.

**Fig 1:**
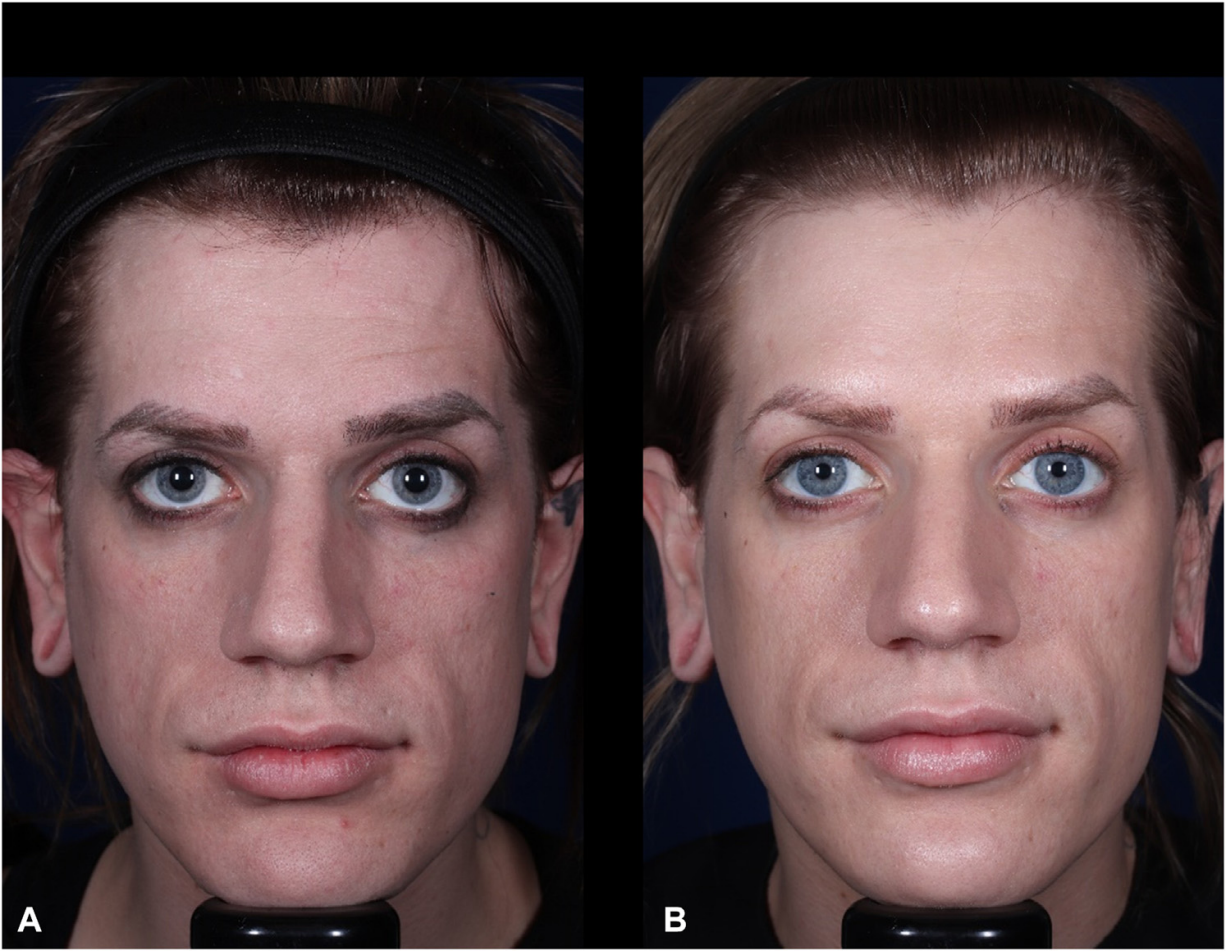
**A,** Image on left was taken at baseline. **B,** Image on right was taken at month 9.

At the 1-month follow-up, the subject’s glabellar line at animation score was graded as 0, indicating the absence of lines and smooth skin. The subject reported being highly satisfied with their appearance, feeling that they appeared significantly younger than their age. Overall, the subject expressed great satisfaction with the aesthetic outcome achieved in the glabella. Additionally, they experienced an enhanced sense of attractiveness and an improved self-image.

At the 3-months postinjection visit, muscle movement began to return. The investigator’s live assessment revealed the presence of mild glabellar lines at animation (grade 1) in the subject. Despite this, the subject remained content with their appearance, believing it reflected their actual age. The subject expressed satisfaction with the aesthetic outcome obtained. Importantly, when engaging in facial expressions such as frowning, concentrating, or showing anger, the subject experienced minimal to no bother from their glabellar lines. Overall, they believed confident, comfortable, and happy with themselves.

At the 6-month milestone, the subject had reverted to baseline conditions for muscle function—and then received a reinjection of 50 U of abobotulinumtoxinA. The effects were similar upon reinjection, displaying improvements at month 1 and muscle function was beginning to return from month 3 to month 6.

At the month 9 visit, however, the subject stated that they underwent cosmetic alterations to their appearance—specifically in the form of breast augmentation surgery (Fig 1). The patient’s gender change was recorded; however, the clinical study used gender at birth for end point data analysis.

This case likely serves as one of the first examples of a subject transitioning in clinical trial and displays the importance of inclusivity and understanding the diverse experiences of subjects in medical research. Trials should indicate birth gender as well as current gender on source documents for each visit. All in all, the subject, at all time points, was pleased with their study treatments and demonstrated substantial 2-grade improvements in their glabellar lines leading to a notable improvement in their self-image, confidence, and overall happiness.

## Conflicts of interest

Dr Cohen is a paid consultant and clinical investigator for Galderma Laboratories, LP. Dr El-Qadi is an employee of Galderma Laboratories, LP.
